# Shear Behavior of Hybrid Fiber Reinforced Concrete Deep Beams

**DOI:** 10.3390/ma11102023

**Published:** 2018-10-18

**Authors:** Kaize Ma, Ting Qi, Huijie Liu, Hongbing Wang

**Affiliations:** Department of Civil Engineering, Chang’an University, 201 Yanta Road, Xi’an 710061, China; qitingtingqi@126.com (T.Q.); 13572176599@163.com (H.L.); whb0414@163.com (H.W.)

**Keywords:** hybrid fiber reinforced concrete, deep beam, shear behavior, tensile strength, ultimate load

## Abstract

Hybrid fiber reinforced concrete (HFRC) is based on a multilevel-reinforcement material design that improves both the compressive strength and tensile strength. Investigations of the mechanical performance of HFRC with two types of steel fibers were conducted experimentally. The investigated parameters were the volume fractions of the short steel fibers and long steel fibers. The compressive strength, tensile strength, and flexural strength of the HFRC were researched. The group with volume fractions of 1.5% for the long steel fibers and 0.5% for the short steel fibers exhibited the best flexural strength. The synergetic effect clearly was improved by combining different types of steel fiber. Four HFRC deep beams and one reinforced concrete (RC) deep beam were conducted to consider the shear behavior of these beams. The primary variables included the volume fraction of steel fibers and the web reinforcement ratio. The shear behavior was evaluated based on the cracking pattern, load-deflection behavior, and shear capacity. All of the beams failed due to the formation of diagonal cracks. The results indicated that hybrid fibers contribute greatly to the shear behavior of deep beams. The hybrid fibers led to the formation of multiple diagonal cracks in the deep beams and enhanced the damage tolerance. With the same web reinforcement ratio, the ultimate load and deformation of the HFRC deep beams were better than those of the RC deep beam.

## 1. Introduction

Fiber reinforced concrete (FRC) is commonly used in civil engineering construction, such as in the production of slabs, industrial floors and precast concrete products [1,2,3,4]. The incorporation of fibers significantly improves the mechanical performance, including the tensile strength, fracture toughness, and fatigue resistance of the reinforced concrete. The fibers serve as not only the cracking control reinforcement, but also the vehicle to allow for significant internal plastic stress redistribution to increase the strength of the specimens after the first crack forms [5,6,7,8,9,10,11].

Hybrid fiber reinforced concrete (HFRC) is a material that contains different types of fibers, like steel fibers or PVA fibers or different shapes and geometry of steel fibers [12,13,14] applied a variety of different types of fibers in a concrete mixture and advised multimodal fiber reinforced concrete. Many researchers further developed the HFRC concepts [15,16]. The hybrid steel fibers can effectively bridge cracks at both the micro- and macroscale, and the cracking of concrete is a multiscale and multistage process. Hybrid fibers with different dimensions are usually used to restrain different scales of cracks during the loading process. The microfibers provide effective reinforcement of the micro cracking stage and inhibited the formation of microcracks. The HFRC shows good performance in terms of the confinement and tension stiffening effects. In addition, HFRC also exhibits desirable crack damage tolerance characteristics with multiple fine cracks [17,18,19]. The control of microcracks is essential for enhancing the durability of RC structures. Because cracking due to expansive deterioration processes initiates as microcracks near the reaction sites, these microcracks need to be controlled at the onset before they become macrocracks [20,21,22].

Deep beams with a small shear span to effective depth ratio could carry much greater shear force; these beams are used in building structures, such as transfer girders, folded plates and cap beams. The failure mode of deep beams was usually shear failure rather than flexure failure. It has been widely shown that the shear capacity of RC deep beams increased as the strength of the concrete and web reinforcement ratio increased [23,24,25]. FRC deep beams exhibit smaller crack widths and higher ultimate loads than those of RC deep beams [26,27,28,29,30] researched the flexural behavior of RC and FRC deep beams. The experimental results indicated that the brittle behavior of RC beams was mainly manifested in the crushing of concrete struts and the fracturing of web reinforcements. The FRC deep beams showed a higher shear capacity due to the effect of the fibers. Refs. [31,32] investigated the influence of steel fiber reinforced concrete deep beams with openings. The results showed that a significant final deflection and a notable portion of the shear capacity were maintained.

In this research, HFRC with two different fiber sizes and various volume fractions were add to a high strength concrete mix to study the flowability, compressive strength, tensile strength, and flexural strength of the HFRC. The failure mechanism, shear capacity, and ductility of HFRC deep beams were investigated. The objective of the research was the advantageous effect of hybrid materials improving the shear behavior of deep beam.

## 2. Material Investigated

### 2.1. Material Proportions

**Figure 1:**
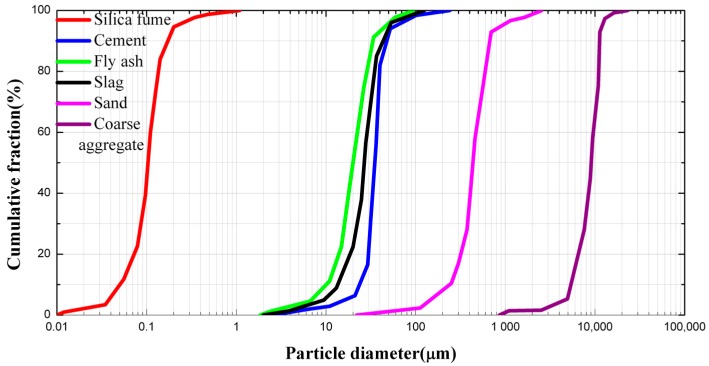
Particle size distribution of the used material.

**Figure 2:**
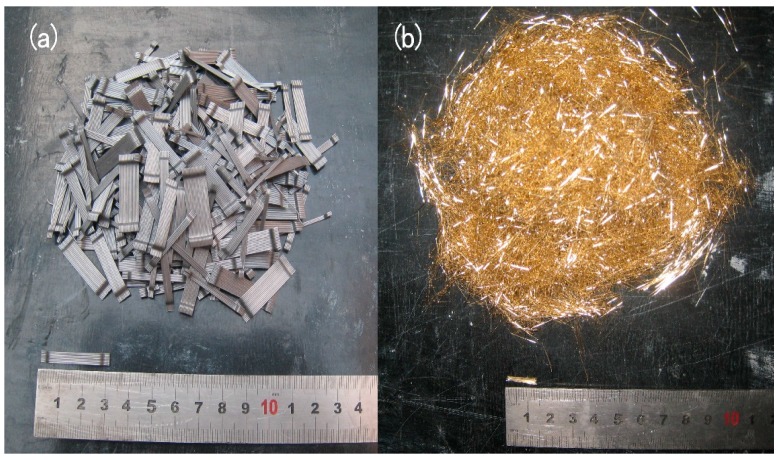
Steel fibers of (**a**) straight fiber and (**b**) hooked end fiber.

The details of the mixture proportions are summarized in Table 1. Portland cement I 52.5R (Hailuo, Xi’an, China) was used, with a 28-day compressive strength of 58.5 MPa. Supplementary cementitious materials, such as fly ash, silica fume, and ground blast furnace slag were used due to both their technical and economic advantages. In general, smaller aggregate will result in higher compressive strengths, but the use of large aggregate allows for a higher Young’s modulus and a better creep and shrinkage behavior. The maximum diameter of the coarse aggregate was 16 mm. River sand was utilized as the fine aggregate, and the maximum diameter and fineness modulus were 5 mm and 2.83 mm, respectively. The particle size distribution of the materials used is shown in Figure 1. A high water/binder ratio was detrimental to the mechanical properties, deformation, and durability of the concrete. However, a low water/binder ratio was detrimental to the flowability. The water/binder ratio of the HFRC was 0.24. The use of superplasticizers was essential to achieve high strength and good flowability. Two types of fibers with different sizes were added to the test groups, and the specific mechanical properties of fibers are summarized in Figure 2 and Table 2. 

### 2.2. Material Fabrication

To produce flowable HFRC, the cement, silica fume, fly ash, slag, fine river sand, and coarse aggregate were premixed for three minutes. Then, water mixed with superplasticizer was poured into the dry mixture for three minutes. When the mixture reached a satisfactory viscosity and fluidity, the hybrid steel fibers were evenly mixed into the mixture. Next, the mixture was mixed again for three minutes. The HFRC was cast into molds and vibrated with a frequency vibrator for one minute. The HFRC specimens were removed from the mold after 24 h and then placed in a standard curing room with a temperature and humidity of 20 ± 2 °C and ≥95% for 27 days.

### 2.3. Flowability Test

**Figure 3:**
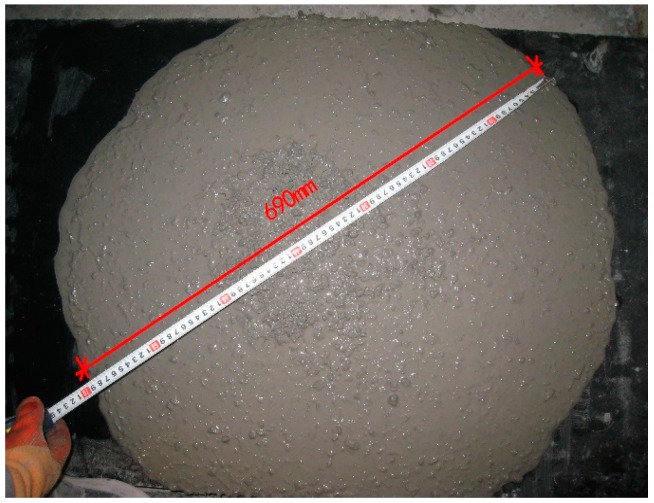
Flow ability.

The flowability tests of the HFRC were evaluated with slump ASTM C143 [33] and slump flow ASTM C1611 [34]. The results were given in Table 3 and Figure 3. Although the HFRC had less flowability than the plain concrete, it was generally proven that the dispersion of the fibers was satisfactory, the fibers were found to be uniform and there was no significant balling. For a volume fraction of long steel fibers of 1%, the slump of HFRC1 was 259 mm. When the volume fraction of the long steel fibers was 2%, the slump was 235 mm. When the volume fraction of the long steel fibers increased to 3%, the slump sharply decreased to 173 mm. The slump flow of groups HFRC2, HFRC5, HFRC6, and HFRC7 (each of which have the same fiber volume fraction of 2%) decreased as the volume fraction of short steel fibers increased. When comparing groups HFRC2 with HFRC7, the slump flow decreased 22.4%. The flowability was considerably influenced by the increase in the volume fraction of short steel fibers. 

### 2.4. Compressive Test

**Figure 4:**
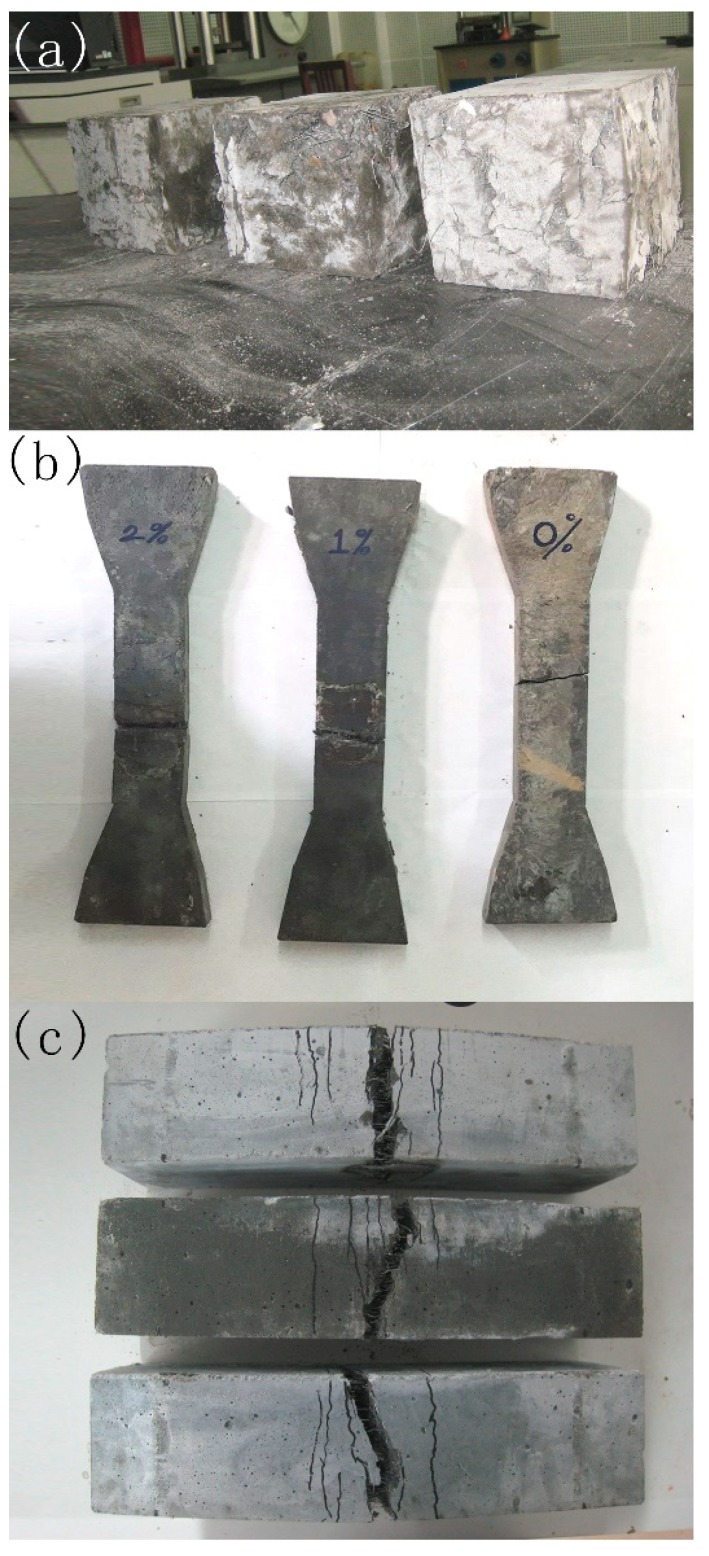
Failure model; (**a**) compression; (**b**) tension; and (**c**) flexure.

For the compressive tests, the cubic specimens had a side length of 150 mm; the specimens had dimensions of 150 mm × 150 mm × 150 mm. The load was applied with a universal testing machine (UTM) (CIMACH, Changchun, China) and the value of the maximum load capacity of the UTM was 2000 kN. An axial compressive loading was applied to these cubic specimens at a rate of 0.05 mm/min. For the HFRC cube, several microcracks appeared as the load increased up to 70–75% of the compressive strength. After cracking, the stiffness of the compression curve decreased progressively. At the maximum load, the microcracks transformed into macrocracks. The failure presented a ductility behavior due to the presence of steel fibers. The compressive strengths of the mixtures are shown in Table 3, and failure modes are shown in Figure 4. The HFRC had a higher compressive strength than that of plain concrete, and the rate of increase ranged from 5.3% to 37.5%. For the specimens with a volume fraction of steel fiber of 2%, the compression of the specimens showed a better performance when short steel fibers were added due to the defect improvement, which were evenly dispersed. The increase in compressive strength was approximately 10.8% as the volume fraction of short fibers was increased from 0% to 2%.

### 2.5. Tensile Test

**Figure 5:**
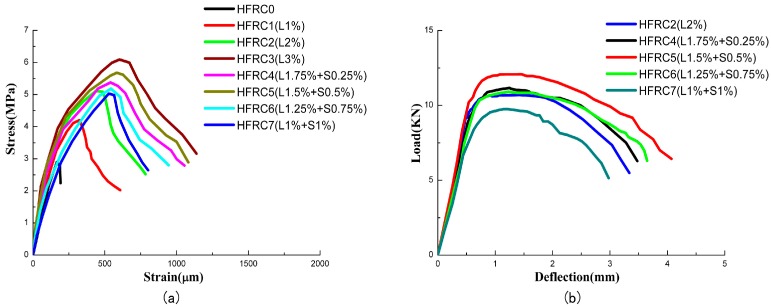
Load-deflection curves. (**a**) tensile test; and (**b**) flexural test.

The direct tensile test specimens with dimensions of 300 mm × 50 mm × 25 mm were used. The load was applied with a universal testing machine (UTM) (CIMACH, Changchun, China), and the value of the maximum load capacity of the UTM was 100 kN. The tests were displacement control at a rate of 0.05 mm/min. A set of mechanical grips were used and the tensile load and actuator displacement were recorded. The stress-strain curves of different groups are shown in Figure 5a. Table 3 presents the direct tensile strengths of all the mixtures. The deflection hardening response with multiple microcracks led to the growth of cracks. The tensile strength of the HFRC was significantly affected by the addition of fibers. Comparing groups HFRC1, HFRC2, and HFRC3 with group HFRC0, the tensile strength increased 34.6%, 75%, and 109.6%, respectively. As shown in the direct tensile tests, the long steel fibers greatly contributed to the strength. The HFRC had a higher tensile strength than that of the concrete with a single type of fiber. The tension-compressive strength ratio ranged from 1/27.5 to 1/16.3. The volume fraction of the short steel fibers increased from 0 to 0.5%, and the tensile strength was improved. The strengthening effect was maximized when the volume fraction of the short steel fibers was 0.5%. The tensile strength of HFRC7, mixed with 1% short fibers, was lower than that of HFRC2. The highest tensile strength was achieved by the groups with a volume fraction of long steel fibers of 1.5% and short steel fibers of 0.5%. 

### 2.6. Flexural Test

HFRC beams with dimensions of 100 mm × 100 mm × 400 mm were fabricated for the four-point flexural tests, which were carried out in accordance with ASTM C1609-12 [35]. A UTM (CIMACH, Changchun, China) with a maximum load capacity of 300 kN was used, and an axial load was applied with displacement control using at a rate of 0.1 mm/min. Each beam was loaded under four-point flexure to create a pure moment region spanning 100 mm at the center of the beam. The load-deflection curves of different groups are shown in Figure 4. For example, HFRC4 can be used to demonstrate the damage process. Before cracking, the curve of the specimen was approximately linear. After cracking, the loading stiffness decreased, and many flexural cracks appeared in the middle of the HFRC beam. The HFRC beams exhibited a deflection hardening behavior. The short steel fibers bridged the microcracks and improved the cracking distribution by reducing the cracks spacing. The influence of the short fibers decreased as the width of the cracks increased. The long fibers were responsible for the hardening phase. At the ultimate load, the major flexural crack formed. The fibers were pulled out, and squeaking was heard. Finally, the beam failed in flexural failure. The flexural strength is reported in Table 3. The HFRC beams exhibited ductility in the flexural test. The addition of steel fibers into normal concrete mixtures significantly improved the flexural strength of the beams. Compared with HFRC0, the post-cracking strength of HFRC1, HFRC2, and HFRC3 increased by 22.4%, 43.5%, and 116.6%, respectively. It was observed that the flexural strength of HFRC3 was higher than that of HFRC2 and increased by 18.4%. When the volume fraction of short steel fibers was greater than 0.5%, the flexural strength decreased. The post-cracking tensile strength of flexural beams was computed as $PL/B_{f}^{3}$, where L was the effective length of the beams and $B_{f}$ was the side length of a beam cross-section.

## 3. Experimental Investigation of HFRC Deep Beams

### 3.1. Detail of Deep Beams

**Figure 6:**
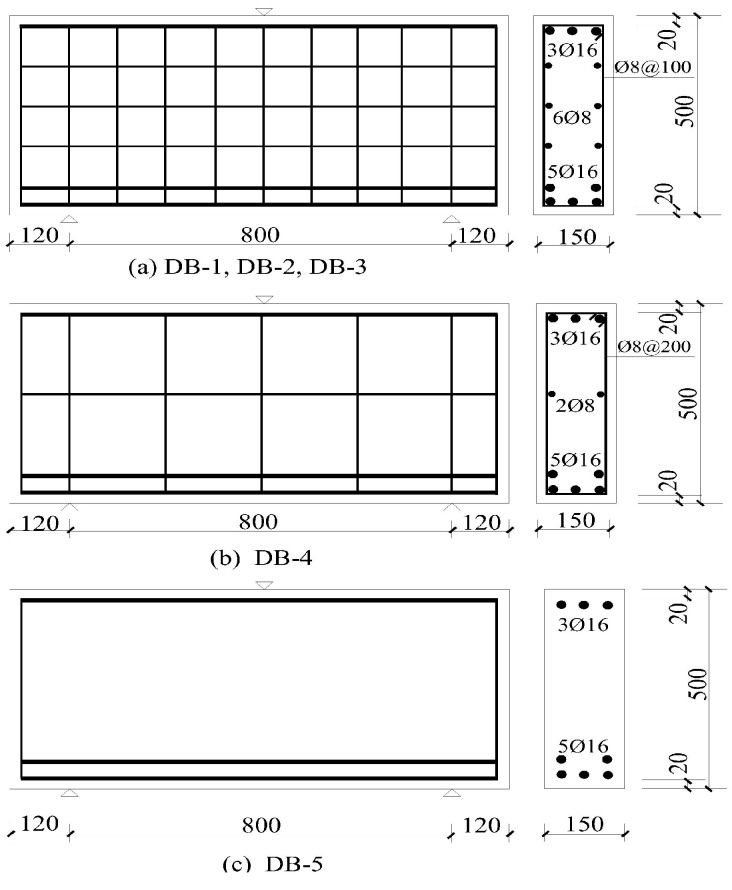
Reinforcement configuration of specimens. (**a**) DB-1, DB-2, DB-3; (**b**) DB-4; (**c**) DB-5.

Four HFRC deep beams and one RC deep beam were tested. The test variables included the fiber volume fractions and web reinforcement ratio. Three different levels were considered for the test variables. All of the test specimens had the same rectangular section with a dimension of 150 mm (width) × 500 mm (height) × 1040 mm (length), and the effective span was 800 mm. The shear span to effective depth ratio (*a/d*) was 0.9. The deep beam reinforcements are exhibited in Figure 6.

### 3.2. Material Properties

The compressive strength, tensile strength, flexural strength and Young’s modulus of the concrete specimens are shown in Table 4. Reinforcements conforming to D16 (16 mm diameter) were used for the longitudinal reinforcements, while those conforming to D8 (8 mm diameter) were used for the stirrups. For each type of reinforcements, three standard test samples were prepared for tensile testing. The means of the yield strength and ultimate strength for the reinforcement of D16 were 472 MPa and 617 MPa, and the corresponding strengths for the reinforcement of D8 were 568 MPa and 723 MPa, respectively. All of the deep beams were cast and cured under the same conditions. All of the specimens were demolded after two days. Then, the specimens were kept wet and covered by a polyethylene sheet until 24 hours before testing.

### 3.3. Test Content and Layout

**Figure 7:**
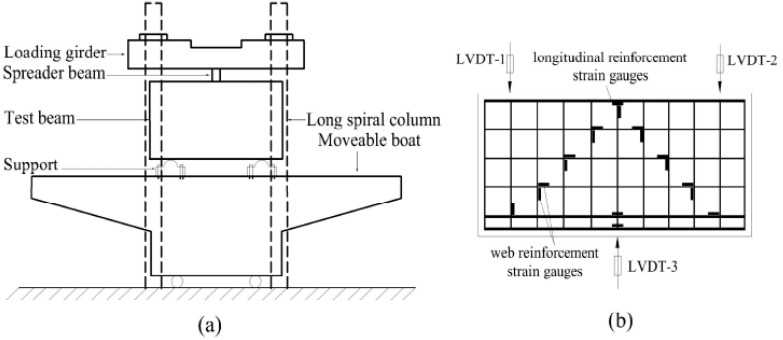
Instrument of test (**a**) setup; (**b**) strain gauges and LVDTs arrangement.

The load value, deflection, reinforcement strains, concrete strains, and crack widths were measured. All the specimens were tested by a 5000 kN hydraulic servo testing machine (MTS Systems Corporation, Eden Prairie, MN, USA) with a monotonically increased loading in the mid-span of the deep beams. The relevant details are shown in Figure 7a. Ten concrete strain gauges were placed on the surface of the concrete in the shear span of the beams perpendicular to the potential direction of a diagonal shear crack, and twelve strain gauges were attached to the longitudinal reinforcement and web reinforcement. The vertical deflection was measured by linear variable differential transformers (LVDTs). Three LVDTs were placed under the specimen, and two LVDTs were placed above the specimen. The arrangement of the strain gauges and LVDTs is shown in Figure 7b. The vertical load was controlled with displacement and the loading rate was 0.1 mm/min. The deep beams were preloaded to check the instrument measurements. A crack-observation instrument was used to measure the width of the cracks. All the test data were collected using a DH3816N data-acquisition system (CIMACH, Changchun, China).

## 4. Results and Discussion

### 4.1. Crack Patterns

**Figure 8:**
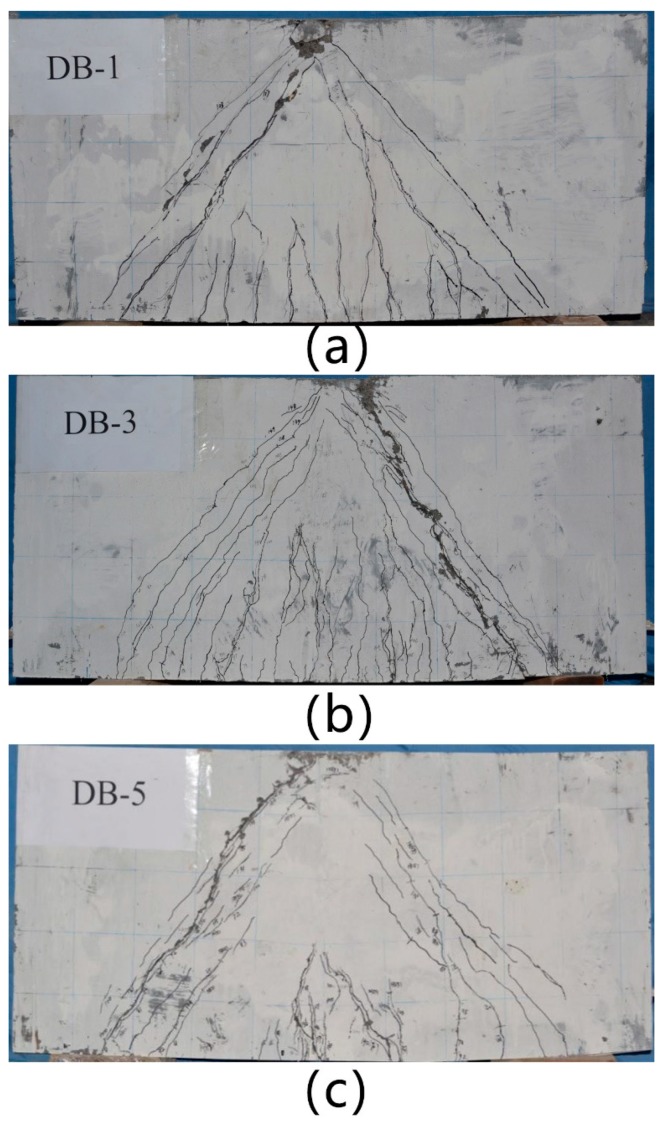
Cracks of specimens. (**a**) DB-1; (**b**) DB-2; (**c**) DB-3.

Regarding specimen DB-1, as shown in Figure 8a, when the load was 180 kN, the first flexural crack appeared across the middle of the beam. As the load increased to 301 kN, the first diagonal crack appeared between the left bearing and the loading point. With increasing load, the diagonal cracks extended quickly. At the ultimate load, the major diagonal crack formed and developed a large crack width. During the unloading stage, the main shear cracks were observed on the left side. The widths of the main shear cracks increased continuously until the experiment stopped. 

Regarding specimen DB-2, when the initial load was 657 kN, the first shear crack appeared at the connection between the right bearing and the loading point. When the load increased, many diagonal shear cracks appeared, and the width of the shear cracks on the right side expanded slowly. During the crack width development, steel fibers were pulled out. The fibers became exposed across the cracks and a loud sound was emitted. At the ultimate load, the major diagonal crack formed. The cracks did not continue increasing uncontrollably due to the presence of the steel fibers. The width of the diagonal cracks clearly increased after the ultimate load was reached. Finally, the deep beam failed in a shear failure mode. 

Regarding specimen DB-3, as shown in Figure 8b, the shear crack appeared at the mid-depth of the beam on every web region of the shear span as the load increased to 844 kN. The diagonal cracks increased and developed toward the shear span. As the volume fraction of steel fibers increased, the number of cracks increased. However, the widths of the cracks decreased. As the load increased from 80% to 90% of the ultimate load, one of the diagonal cracks began to grow wider. Pullout of the steel fibers was also observed. Finally, the deflection of the deep beam increased substantially and reached failure.

Regarding specimen DB-4, when the deep beam was loaded to 392 kN, cracks formed in the vertical direction. With loading up to 761 kN, the shear cracks appeared on both sides of the deep beam. The development of cracks mainly concentrated on the shear region. New small cracks continued to appear and were stably extended. With a continuous increase in the loading, the cracks continued to form. The critical crack was observed on the right side when the ultimate load was reached. The steel fibers were pulled out more intensely and the main crack continued to widen. The load decreased to the ultimate load of 85%, and the test was completed.

Regarding specimen DB-5, as shown in Figure 8c, when the load reached 369 kN, the first flexural crack occurred in the mid-span. As the load increased to 685 kN, a small crack appeared between the left bearing and loading point. Although this specimen did not have web reinforcement, the load slowly decreased after the ultimate load. In addition, the left shear cracks continued to expand. The test was completed when the load decreased to 85% of the ultimate load. In this case, all of the specimens failed in shear.

The experimental results showed that the HFRC deep beams failed in the shear failure mode. When the shear cracks of DB-1 appeared, the width of the cracks quickly increased. With the presence of steel fibers, the shear cracks of DB-2 and DB-3 appeared later than those of DB-1. In other words, the HFRC deep beams were subjected to a higher load when the first shear cracks developed. Once shear cracks appeared in the HFRC deep beams, the steel fibers transferred tension across the cracks, controlled the opening of the cracks and helped maintain aggregate interlocking. Multiple diagonal cracks were observed in the HFRC deep beams. As the test terminated, the number of cracks in the RC deep beam was less than that in the HFRC deep beams. The fibers bridged the cracks and played a major role in the tensile behavior. The steel fibers became effective after the formation of shear cracks and continued to resist the principal tensile stresses and complete pull out. The spacing of the cracks in the HFRC deep beams were observed to be smaller than that in the RC beam due to the uniform redistribution of stress.

### 4.2. Load-Deflection Behavior

**Figure 9:**
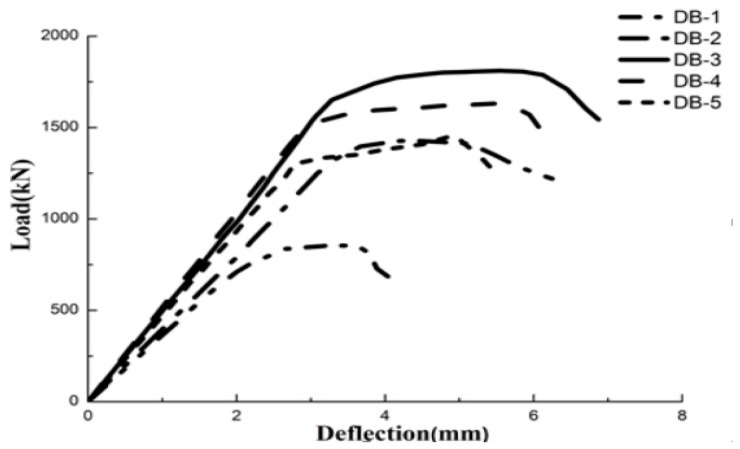
Relationship between the load and mid-deflection.

The curves of the axial load versus mid-span deflections for five of the HFRC deep beams are shown in Figure 9. As expected, the initial stiffnesses of the specimens are similar, depending on the shear span to effective depth ratio. Before the shear cracks appeared, the initial stiffness of all the specimens was constant, and the stiffness increased linearly. The web reinforcement ratio did not affect the first shear cracking load. After the first shear diagonal crack appeared, the RC deep beam stiffness decreased significantly. However, the stiffness of the HFRC deep beams decreased slowly with very small increase in the width of the cracks. No significant stiffness reduction was observed after the formation of the cracks.

In the unloading stage, the descending rates of the HFRC deep beams were slower than that of the RC deep beam. The stiffness decreased quickly after the web reinforcement yielded in the deep beam with a low web reinforcement ratio. Due to the excellent resistance of the HFRC, a high stiffness was exhibited in the load-deflection curves of the HFRC deep beams. 

### 4.3. Ultimate Load and Deflection

The crack load and ultimate load of each specimen are shown in Table 5. Increasing the fiber volume fraction increased both the cracking and the ultimate load of the beams. The ultimate load of DB-2 and DB-3 increased by 66.8% and 114.2% as compared with that of DB-1. The web reinforcement ratios have less of an effect on the ultimate load of the deep beams than that of the fiber volume fractions. For instance, the ultimate load of DB-4 and DB-5 decreased by 9.9% and 19.6% when compared with that of DB-3. A comparison of the test results of DB-5 and DB-1 indicates that the use of steel fibers enhanced the ultimate load by 70.1%, even with a lower web reinforcement ratio.

The ultimate deflection was computed as the load decreased to 85% of the ultimate load. Table 5 summarizes the ultimate deflection of all the test specimens. All of the HFRC deep beams exhibited an improved deformation capacity. The specimen with a reduced web reinforcement due to the use of HFRC bore more load and achieved a sufficient ductility.

### 4.4. Strains in Reinforcement and Concrete

**Figure 10:**
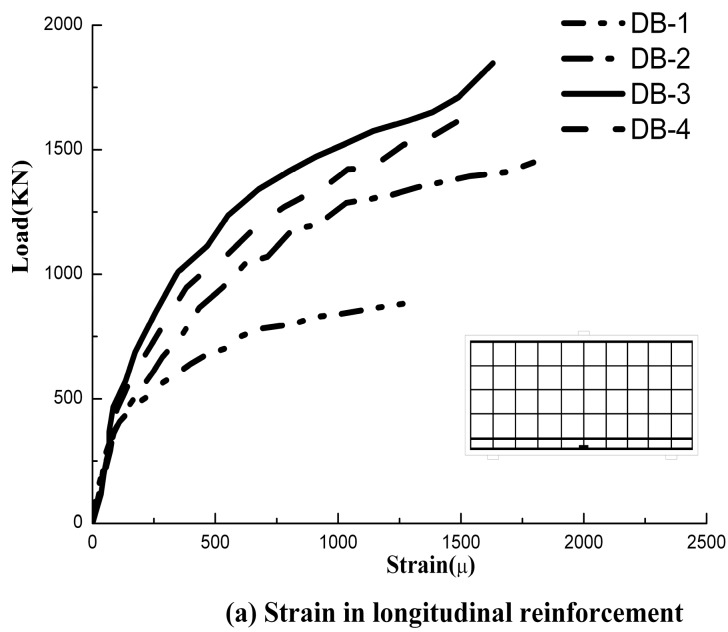

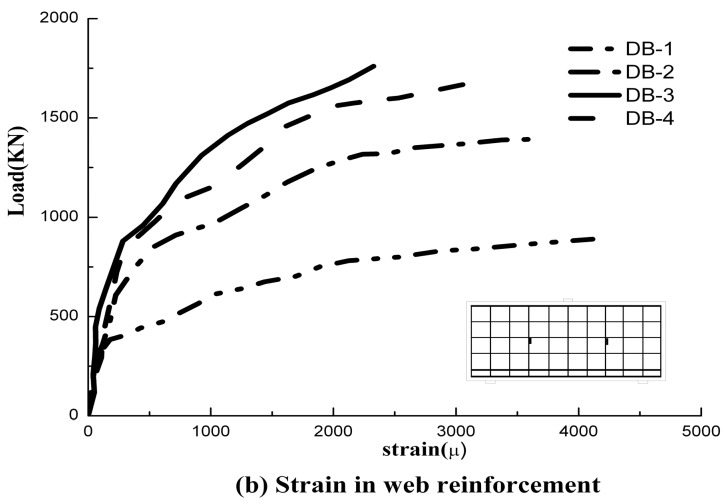
Applied load against strain. (**a**) Strain in longitudinal reinforcement; (**b**) Strain in web reinforcement.

The relationship between load and reinforcement strains is illustrated in Figure 10. The number of strain gauges, which were used in the beams, was decided according to the amount of web reinforcement. All of the strain gauges used in the beams were attached to the web reinforcement in the shear spans. As the first diagonal crack appeared, the concrete strains and web reinforcement developed significantly for DB-1. The presence of the fibers played a major role in the tensile behavior, and the strains of the HFRC deep beams increased slowly. For the deep beams, the largest strains were observed in the web reinforcement across the main diagonal crack that formed between the bearing and loading point. The majority of the web reinforcement reaches the yield strain. However, the strain of the longitudinal reinforcement in the bottom of the beam did not reach the yield strain. In some cases, the position of the strain gauges and major diagonal crack may be biased. Thus, the reinforcement could have yielded even if it was not recorded by the strain gauges.

For the loading of HFRC deep beams, shear forces are transferred through the compression struts to the supports. Irreversible compressive strain accumulates due to the induced compressive stress with the shear span. To maintain equilibrium, the horizontal tensile forces are resisted by the longitudinal reinforcement. With the addition of steel fibers to the beams reinforced with the reinforcement, the shear span deformations (shear strains) were observed to reduce as the steel fibers volume fraction increased from 0% to 2%. This improvement was as a result of crack-bridging of the inclined cracks the shear span, hence retarding the shear strains. 

### 4.5. Discussion

The production of hybrid fibers reinforced s concretes aims to combine the mechanical properties of two or more different fibers. Hybrid reinforcement systems can be used in order to take advantage of each individual fiber properties, which depend on the shape, type, size, and the volume fraction of the used fibers. The principle of long steel fibers and short steel fibers reinforced high-strength concrete is to mix two types of steel fibers into high strength concrete to exert its synergistic effect and enhance or improve some properties of the original single-type steel fiber concrete. When steel fibers of different sizes are mixed, the short fibers, as the “micro reinforcement”, mainly play the important role in strengthening the cement matrix and delaying the expansion of micro cracks. The crack resistances of deep beams were improved effectively. When the large cracks occurred in shear span, the more energy would be dissipated due to the present of the long fibers, which have excellent pullout resistance. The expansion of cracks is delayed and the bearing capacity is improved for HFRC deep beams. Both of short fibers and long fibers complement each other during the failure process of deep beam, which effectively improved bearing capacity and deformation capacity. The HFRC deep beams showed good transformation, allowing for a better transmission of the internal forces. The hybrid steel fibers not only improve the ultimate load, but also control the development of cracks of deep beams. The amount of stirrups required was reduced with the use of steel fibers. The combination of stirrups and fibers could satisfy the shear capacity and deformation requirements.

## 5. Summary and Conclusions

This paper presents research on the mechanical performance of hybrid fiber reinforced concrete. The shear behavior of four HFRC deep beams was investigated and presented. The test variables included the volume fraction of steel fibers and web reinforcement ratio. Based on the results, the following conclusions can be drawn:

(1) The flowability was considerably influenced by the increase in the volume fraction of short steel fibers. The compressive strength of the specimens showed a better performance when short steel fibers were added due to the defect improvement The HFRC had high tensile strength and flexural toughness as the steel volume fractions up to 2%. Adding 1.5% long steel fibers and 0.5% short steel fibers achieved the best toughening effect.

(2) The failure mode of the specimens was mainly diagonal cracking between the bearing and loading point. The numbers of cracks obviously improve. The HFRC substantially limited the extension of the cracks, and reached a higher ultimate load than those of the RC deep beam. 

(3) The ultimate load of DB-2 and DB-3 improved by 66.8% and 114.2%, respectively, as compared with that of DB-1. The ultimate deflection of HFRC deep beams are improved with the increases of the volume friction of steel fibers. The concrete strain and the web reinforcement crossing the diagonal cracks reaches the yield strain in the shear span region. The behavior of the cracking was changed due to bridging fibers across the cracks.

## Figures and Tables

**Table 1:** Material proportions.

Groups	Cement (kg/m^3^)	Silica (kg/m^3^)	Fly Ash (kg/m^3^)	Slag (kg/m^3^)	Fine Sand (kg/m^3^)	Coarse Aggregate (kg/m^3^)	Water (kg/m^3^)	Super Plasticizer (kg/m^3^)	Fibers Volumes (L, S) (%)
HFRC0	325	32.5	195	97.5	722	868	160	1.6	0, 0
HFRC1	325	32.5	195	97.5	722	868	160	1.6	1, 0
HFRC2	325	32.5	195	97.5	722	868	160	2.4	2, 0
HFRC3	325	32.5	195	97.5	722	868	160	2.4	3, 0
HFRC4	325	32.5	195	97.5	722	868	160	2.4	1.75, 0.25
HFRC5	325	32.5	195	97.5	722	868	160	2.4	1.5, 0.5
HFRC6	325	32.5	195	97.5	722	868	160	2.4	1.25, 0.75
HFRC7	325	32.5	195	97.5	722	868	160	1.0	1, 1

**Table 2:** Mechanical properties of steel fibers.

Types	*l_f_* (mm)	*d_f_* (mm)	*E_f_* (GP)	Tensile Strength (MPa)	Shape
Long steel fiber	30	0.55	210	1345	Hook
Short steel fiber	13	0.20	210	2000	Straight

*l_f_* is the length of steel fiber, *d_f_* is the diameter of steel fiber, *E_f_* is the elastic modulus of fiber.

**Table 3:** Material test results.

Groups	Slump (mm)	Slump Flow (mm)	Compressive Strength (MPa)	Tensile Strength (MPa)	Flexural Strength (MPa)
HFRC0	270	720	80.3	2.92	3.95
HFRC1	259	690	84.4	3.93	7.57
HFRC2	235	590	92.6	5.11	10.71
HFRC3	173	435	99.5	6.12	13.99
HFRC4	222	562	93.0	5.45	11.16
HFRC5	210	533	99.8	5.78	12.08
HFRC6	199	497	101.4	5.23	10.83
HFRC7	181	458	103.8	5.02	9.75

**Table 4:** Material properties of concrete.

**Specimens**	**Fibers Volumes (L, S) (%)**	$f_{c}\;(\mathrm{MPa})$	$f_{c}^{\prime }\;(\mathrm{MPa})$	$f_{t}\;(\mathrm{MPa})$	$f_{spt}\;(\mathrm{MPa})$	$E_{c}\;(N/{\mathrm{mm}}^{2})$
DB-1	0, 0	66.2	56.2	3.05	3.05	3.56 × 10^4^
DB-2	0.75, 0.25	68.2	57.3	3.85	6.92	3.63 × 10^4^
DB-3	1.5, 0.5	71.7	60.8	5.22	10.23	3.82 × 10^4^
DB-4	1.5, 0.5	72.1	61.1	5.29	10.42	3.89 × 10^4^
DB-5	1.5, 0.5	71.5	60.5	5.34	10.35	3.81 × 10^4^

$f_{c}$ is cube compressive strength of fiber concrete; $f_{c}^{\prime }$ is cylinder compressive strength of concrete; $f_{t}$ is direct tension strength of concrete; $f_{spt}$ is split tensile strength of concrete; $E_{c}$ is the elastic modulus of concrete.

**Table 5:** Experimental results.

Specimens	$V_{cr}\;(\mathrm{kN})$	$V_{u}\;(\mathrm{kN})$	$\Delta_{u}\;(\mathrm{mm})$
DB-1	301.3	857.2	3.8
DB-2	657.2	1430.2	6.0
DB-3	844.3	1815.0	6.7
DB-4	761.2	1634.9	6.1
DB-5	685.4	1458.4	5.2

$V_{cr}$ is crack load, $V_{u}$ is ultimate load, $\Delta_{u}$ is ultimate displacement corresponds to the load decreased to 85% of the ultimate load.
